# The health beliefs of mothers about preventing cervical cancer and their intention to recommend the Pap test to their daughters: a cross-sectional survey

**DOI:** 10.1186/s12889-016-3037-6

**Published:** 2016-05-03

**Authors:** Hae Won Kim

**Affiliations:** The Research Institute of Nursing Science, College of Nursing, Seoul National University, Taehakro 103, Jongro-Gu, Seoul, 110-799 South Korea

**Keywords:** Cervical cancer, Pap test, Human papillomavirus, Mothers, Adolescents, Korea

## Abstract

**Background:**

Mothers have a primary role in the prevention of cervical cancer in Korea. This study aimed to determine the awareness and health beliefs of mothers about preventing cervical cancer in their daughters, their intention to recommend the Pap test to their daughters, and the factors influencing this intention.

**Methods:**

A cross-sectional survey design was employed, and the study enrolled mothers (*n* = 1,581) of pubescent girls aged 13 to 18 years who were living nationwide in Korea. The six health-beliefs variables related to preventing cervical cancer in their daughters, awareness of the importance of cervical cancer prevention methods, and the intention to recommend the Pap test to daughters were investigated. The impacts of these health beliefs of the mothers and the sociodemographic factors influencing their intention to recommend the Pap test to their daughters were assessed using multiple logistic regression analysis.

**Results:**

Almost one-quarter (23.7 %) of the mothers had talked about the Pap test, 69.2 % were intending to recommend the Pap test to their daughters, and 38.5 % considered that the Pap test could be necessary if their daughters became sexually active. The significant health beliefs influencing the intention to recommend the Pap test were the perceived barriers [odds ratio (OR) = 1.47, 95 % confidence interval (95 % CI) = 1.03–2.11] and benefits (OR = 2.25, 95 % CI = 1.55–3.25). The significant sociodemographic factors of mothers were their education (OR = 1.52, 95 % CI = 1.08–2.13), their experience of talking about the Pap test with their daughters (OR = 2.11, 95 % CI = 1.23–3.64), their regularity of undergoing the Pap test themselves (OR = 1.98, 95 % CI = 1.30–3.03), and their age when they first underwent the Pap test (OR = 1.60, 95 % CI = 1.43–0.82).

**Conclusions:**

The mothers perceived HPV vaccination as the most important of the five methods for preventing cervical cancer in their daughters. Mothers perceived the importance of their daughters undergoing the Pap test regardless of the presence of HPV vaccination, and most of the mothers had an intention of recommending the Pap test to their daughters. Strategies for increasing the intention of mothers to recommend the Pap test to their adolescent daughters could be promoted by increasing their perceptions of the benefits while reducing their perceptions of barriers toward their daughters undergoing the Pap test, and by empowering active communication about the Pap test between mothers and daughters.

**Electronic supplementary material:**

The online version of this article (doi:10.1186/s12889-016-3037-6) contains supplementary material, which is available to authorized users.

## Background

The incidence rate of cervical cancer (calculated using age-standardized data) in Korea was 11.1 per 100,000 persons in 2013 [1], and in 2013 cervical cancer was the third most prevalent form of cancer among females aged 15–34 years (5.5 per 100,000 persons), after thyroid cancer and breast cancer (71.7 and 10.6 per 100,000 persons, respectively) [1]. This means that young women should be a focus of attempts to prevent cervical cancer in Korea [2–4].

It is generally well known that the primary method for preventing cervical cancer is the human papillomavirus (HPV) vaccination at a prepubertal age [5] and that the secondary prevention method is Papanicolau (Pap) screening for sexually active women [6]. Currently the HPV vaccination is not widely implemented in Korea because there is no mandatory vaccination program and a payment is required when vaccinating adolescents against HPV [7, 8]. The rate of HPV vaccination has recently been reported to be 9.5 % among pubescent girls [9] and 5 % among university students in Korea [4]. At present the primary strategy for preventing cervical cancer among young women in Korea involves the promotion of Pap testing [2, 4, 10]. Whether the Pap test should be encouraged among adolescent girls remains controversial; delaying the Pap test until 21 years of age has been supported by the probability of getting cervical cancer reportedly being low among adolescents in Western countries [11, 12]. The Committee for Cervical Cancer Screening in Korea recently updated the recommendation made in 2002 in which they confirmed the effectiveness of screening including the Pap test with a moderate level of evidence, and recommended it for women aged 20–74 years based on the increasing trend of cervical cancer in women aged 20–29 years [13]. In this revised recommendation, the age for commencing Pap screening was suggested to be the same as that in 2002 despite the introduction of new methods, such as HPV testing [13]. However, it is reported that the Pap testing rate ranged from 7.1 % (mean age = 26.4 years) to 12.5 % (mean age = 22.4 years) among sexually active women in Korea [4, 10]. Based on this situation, it has been suggested that the importance of Pap testing of young Korean women relative to the HPV vaccination needs to be emphasized to the general population in Korea [2, 4]. HPV infection has been considered an important aspect of the sex education provided to teenagers [5, 14], and so Pap testing should also be recognized as an important method for preventing cervical cancer caused by high-risk HPV infection. Moreover, Korean mothers can play a positive role in preventing cervical cancer in their daughters [2, 15]. Studies involving US and French mothers found that their beliefs about their daughters undergoing the Pap test were significantly associated with their intention to vaccinate their daughters [16, 17]. However, little is known about the impact of the comprehensive beliefs of mothers about preventing cervical cancer in their daughters, including about the Pap test, on their intention to recommend the Pap test. Mothers can have a poor awareness about how to prevent cervical cancer in their daughters [15], which makes it necessary to understand the underlying awareness, perceptions, and role of Korean mothers. Moreover, there is currently no formal sex education provided to Korean pubescent girls on cervical cancer prevention or HPV prevention [2, 7, 8, 18]. To address this situation, the current study investigated the role of mothers in preventing cervical cancer in their adolescent daughters, with a focus on the opinions of mothers about their daughters undergoing the Pap test.

There is considerable evidence from previous studies that the health belief model (HBM) is effective for identifying perceptions about preventing cervical cancer and HPV infection, and predicting the subsequent intention to prevent these conditions [4, 7, 8, 19]. However, the components of the HBM neither have the same construct nor have provided consistent predictions of such an intention [8, 19]. The present study used six health beliefs to assess the perceptions of mothers about preventing cervical cancer in their daughters, and examined the impact of the health beliefs of the mothers on their intention to recommend the Pap test to their daughters. Based on the results of previous studies, the influences of sociodemographic factors associated with intention were also examined in the present study, including income [7], history of the Pap test [7, 20–22], sexual health history [16, 21], and talking about HPV or cervical cancer with daughters [15, 16].

This study aimed to determine the awareness and health beliefs of mothers about preventing cervical cancer in their daughters, their intention to recommend the Pap test to their daughters, and the factors influencing this intention.

## Methods

### Study design

A cross-sectional survey design was employed in this study.

### Subjects and data collection procedure

Mothers of puberty daughters aged 13–18 years living in multiple locations in Korea were recruited to participate in this study. It was considered in this study that the aged 13–18 years are correspond to the puberty or teenage among the adolescents. The early adolescents were not included, because they seem to be less relevant with the Pap test. The exact number of mothers with puberty daughters in Korea could not be determined, and so the national data of the population of married women were nominated as a proxy measure. From the available statistics, the number of married women aged from 30 to 60 years was estimated to be 10,287,076 in Korea in August 2013 [23]. To ensure that the study sample was nationally representative, eight regions covering the entire country were sampled, with convenience sampling then employed in each region. The nominated proxies represented from 3.0 % (Kang Won) to 30 % (Kyung Gi and Incheon) of the total population in each region, and it was assumed that a minimum of 45 mothers in each region needed to be included (corresponding to 3.0 % in this study). Therefore, it was estimated that including 1,485 mothers across the regions would be sufficient. Considering a possible dropout rate of 10 %, 1,633 mothers were required. The final numbers of the participants were 1655. Those participants who did not complete either the questionnaire or written consent form (*n* = 74) were excluded. Ultimately 1,581 Korean mothers were eligible for inclusion in the data analysis performed in this study. The regional stratification of the sample was highly representative of the national distribution according to the eight classified administrative regions of Korea: Seoul = 20.0 % (*n* = 316; actual, 20.0 %), Kyung-Gi and Incheon = 30.5 % (*n* = 483; actual, 30.0 %), Kyung-Buk = 7.5 % (*n* = 118; actual, 10.0 %), Busan and Kyung-Nam = 17.0 % (*n* = 269; actual, 16.0 %), Jeon-Nam and Jeju = 7.8 % (*n* = 123; actual, 7.0 %), Chung-Nam and Daejeon = 7.5 % (*n* = 118; actual, 7.0 %), Chung-Buk and Jeon-Buk = 6.3 % (*n* = 99; actual, 7.0 %), and Kang-Won = 5.0 % (*n* = 56; actual, 3.0 %). Subjects were included if they had a daughter aged 13–18 years, understood the purpose of the study, and willingly agreed to participate. Mothers were excluded if they (a) had a serious physical and psychological illness that prevent them answering the questionnaire, (b) were currently receiving medical treatment for cervical cancer, or (c) were non-Koreans. Potential subjects were evaluated and screened by research assistants prior to data collection. Eight of the 12 research assistants involved in the study were nurses. Prior to conducting the research, a written manual detailing the study procedure was provided to all assistants in order to ensure the equal prepartion, and their suitability as assistants was confirmed by a telephone conversation with the primary investigator about the processes of data collection, sending data to the primary investigator, and selection criteria for the subjects.

The research protocol was approved by the Institutional Review Board of Myoung-Ji University Hospital. The participants were contacted with assistance from community leaders, and data were collected by the 12 research assistants living in their local regions. Each assistant visited the participant’s house or another convenient meeting place after making an appointment to perform data collection. Prior to completing surveys, the subjects were given information regarding the specific study aim, contents, and estimated time to respond to the survey, and were assured that all of the information collected would be processed anonymously and confidentially, after which they read and signed an informed consent form agreeing to their participation in the study.

### Measures

The developed questionnaire was tested in a pretest involving ten mothers of girls, and it was finally adopted after making appropriate corrections. Mothers were asked to respond the following order. An additional survey questionnaire file shows this in more detail [see Additional file 1].

The first, the awareness of the mothers about the importance of each of the following five methods for preventing cervical cancer in their daughters was assessed on a scale ranging from 0 (not at all aware) to 100 (completely aware), which had been applied to unmarried university students in a previous study [2], by asking “How important are the following methods for preventing cervical cancer in your daughter?” with the following items based on the guideline of the Korean National Cancer Center [24]: (1) “Initiating and undergoing the Pap test regularly,” (2) “Abstinence from sexual intercourse until adulthood,” (3) “Preventing sexually transmitted infection if sexually active,” (4) “Using condoms regularly,” and (5) “Receiving an HPV vaccination.”

The second, the health beliefs of mothers about preventing cervical cancer in their daughters, including about the Pap test, were measured using the Korean version of the scale for the beliefs about the Pap test and cervical cancer (KCPC-28) as used in a previous study [4], which had confirmed its validity and reliability among Korean women. For the present study, when mothers were asked about their beliefs using KCPC-28, the questions for the mothers with the “I” perspective were modified into “if it would be my daughter” or “with regard to my daughter.” KCPC-28 is based on HBM variables related to cervical cancer prevention, including the Pap test, comprising the risks of cervical cancer (seven items), barriers to the Pap test (seven items), external pressures to undergo the Pap test (six items), awareness of the Pap test (two items), benefits of the Pap test (three items), and the necessity of the Pap test (three items). All of the variables were assessed on a 4-point scale ranging from 1 (strongly agree) to 4 (strongly disagree). Higher subtotal scores indicated that mothers (1) perceived that cervical cancer represented a lower risk to their daughters, (2) perceived lower barriers to their daughters undergoing the Pap test, (3) agreed less with the influences of other persons and the media on their daughters undergoing the Pap test, and (4) had greater awareness and perceived (5) greater benefits and (6) greater needs of their daughters undergoing the Pap test. The reliabilities of these six subscales in the present study were confirmed by Cronbach’s α values of 0.82, 0.80, 0.83, 0.62, 0.68, and 0.85, respectively. The total Cronbach’s α values of the scale in the current and the previous study of the Korean women [4] were 0.81 and 0.74, respectively.

Prior to asking opinion about the daughters’ Pap test, the following information was given to the subjects prior to asking them their opinion about preventing cervical cancer in their daughters and the Pap test. The information was based on the following national recommendations for early screening of cervical cancer in Korea: “HPV infection can occur by sexual transmission, some types of high-risk HPV cause cervical cancer, and the Pap test is recommended annually for all sexually active women older than 20 years and also for females younger than 20 years if their are suspicions of cervical cancer and a precancerous lesion” [3]. The vaccination against HPV was developed to prevent cervical cancer, and so receiving this vaccination is recommended before the onset of sexual activity [3, 25].

The third, to determine the opinions of the mothers toward their daughters undergoing the Pap test, they were asked “When is the most suitable period for your daughter to undergo her first Pap test?” with the following possible responses: (1) “Prior to adulthood if she is sexually active”, (2) “In adulthood regardless of her sexual activity,” (3) “After she marries,” or (4) “My daughter can decide for herself.”

The fourth, with regard to combining HPV vaccination with the Pap test, the mothers were also asked “If your daughter has already received the HPV vaccination and if she is sexually active, does she also need to undergo the Pap test?” with the following possible responses: (1) “The Pap test is not necessary because she has already received the HPV vaccination”, (2) “I don’t know”, (3) “She needs to undergo the Pap test in adulthood”, or (4) “She needs to undergo the Pap test prior to adulthood even though she has already received the HPV vaccination.” The mothers were then asked about their intention to recommend the Pap test to the daughters if it is considered necessary, with possible responses of “yes” or “no.”

Lastly, sociodemographic factors were collected from the participants, including the mother’s age, education, employment status, monthly household income, religion, whether she had talked about HPV and the Pap test with her daughter, whether her daughter was vaccinated against HPV, and whether she had undergone the Pap test, how old she was when she first underwent the Pap test, whether she had been diagnosed cervical cancer in the past, and whether she had a family history of cervical cancer.

### Statistical analysis

All variables were analyzed using frequencies, means, proportions, standard deviations, and percentages. Odds ratios (ORs) and 95 % confidence intervals (95 % CIs) were calculated to examine the potential associations between the sociodemographic factors, the health beliefs, and intention to recommend the Pap test to their daughters. Then the variables yielding significant associations at *p* < 0.05 were included in a logistic regression analysis to build a model between the dependent variable (intention to recommend the Pap test) and independent variables with a probability cutoff of *p* < 0.05.

The dependent variable was assigned values of 0 and 1 for the no-intention and intention groups, respectively. Among the independent variables, the age at which the mothers first underwent the Pap test was divided into 12–32 years and 33–55 years, since the mean age was around 33 years. Regarding the health beliefs of the mothers about preventing cervical cancer in their daughters, the categorizations were also converted into the following dichotomous scales (ranked as high and low) according to their mean values: scores of 7–14 and 15–26 for the risks of cervical cancer, respectively, 9–20 and 21–28 for barriers to the Pap test, 6–14 and 15–24 for outside pressures to undergo the Pap test, 5–8 and 2–4 for awareness of the Pap test, 6–12 and 3–5 for benefits of the Pap test, and 9–12 and 3–8 for the necessity of the Pap test.

The sociodemographic data were categorized as follows: 31–44 and 45–59 years for age, middle or high school and college or above for education, housewife and part or full time for employment status, and 1,000–3,500 and 3,501–5,000 Korean won (1,000 won is almost equal to 1 USD) for the monthly household income. The other following independent variables were scored as 0 and 1 for “no” and “yes” responses, respectively: religion, “Have talked about HPV with my daughter,” “Have talked about the Pap test with my daughter,” “My daughter has received the HPV vaccination,” “I have been diagnosed with cervical cancer,” and “Family history of cervical cancer.” The levels of “I have undergone the Pap test” were categorized as none, irregularly, or regularly. All statistical analyses were conducted using the IBM SPSS Statistics 20 software (IBM, Armonk, NY, USA).

## Results

### Characteristics of the study sample

The age of the study subjects was 44.4 ± 3.88 years (mean ± SD, range 31–59 years). Of the 1,581 subjects, 44.4 % (*n* = 692) were 40–44 years old, 54.1 % (*n* = 839) were educated at the high school level, 44.1 % (*n* = 684) were housewives, 33.6 % had a family monthly income of 2,001–3,500 Korean won, and 35.8 % (*n* = 563) did not have a religion.

One-third of the mothers had talked about HPV with their daughters, 23.7 % had talked about the Pap test, 7.2 % reported that their daughters had already been vaccinated against HPV, 64.8 % of the mothers had themselves undergone the Pap test, 19.1 % of the mothers underwent the test regularly, 47.6 % of the mothers had first undergone the Pap test at an age of 30–35 years (32.9 ± 6.0 years, *n* = 974), 8.6 % of the mothers had been diagnosed with cervical cancer previously, and 4.6 % of the mothers had a family history of cervical cancer (Table 1).

**Table 1:** Characteristics of the subjects (total *n* = 1581; missing values excluded)

	Categories	*n*	%
Sociodemographic factors			
Age	44.40 ± 3.88 years (mean ± SD, range = 31–59 years)		
	31–39 years	114	7.3
	40–44 years	692	44.4
	45–49 years	601	38.6
	50–59 years	149	9.6
Education	≤Middle school	53	3.5
	High school	839	54.1
	≥College	656	42.4
Employment status	Housewife	684	44.1
	Part time	517	33.4
	Full time	349	22.5
Monthly household income	1000–2000	257	18.2
(Korean won × 1,000)	2001–3500	472	33.6
	3501–4500	296	21.0
	4501–5000	381	27.2
Religion	None	563	35.8
	Christianity	350	22.3
	Catholicism	164	10.4
	Buddhism	410	26.0
	Other	86	5.5
Have talked about HPV with daughter	Yes	518	33.0
	No	1051	67.0
Have talked about the Pap test with daughter	Yes	369	23.7
	No	1191	76.3
Daughter has received HPV vaccination	Yes	112	7.2
	No	1446	92.8
Has undergone the Pap test	Regularly	297	19.1
	Irregularly	712	45.7
	No	548	35.2
Age at first Pap test (*n* = 974)	32.9 ± 6.0 years (mean ± SD, range = 12–55 years)		
	12–29 years	208	21.3
	30–35 years	463	47.6
	36–55 years	303	31.0
History of cervical cancer diagnosis	Yes	136	8.6
	No	1442	91.4
Family history of cervical cancer	Yes	73	4.6
	No	1508	95.4

*HPV* human papillomavirus

### Awareness of mothers about methods for preventing cervical cancer in their daughters, including the Pap test

Among the five methods for preventing cervical cancer in their daughters, mothers ranked the importance of undergoing a regular Pap test in second place (score, 78.4 ± 21.4; possible range, 0–100), after HPV vaccination (score, 85.2 ± 16.6). They ranked abstinence from sexual intercourse until adulthood as having the lowest importance (score, 73.3 ± 27.9). Regarding the most suitable time for when their daughters should first undergo the Pap test, 38.5 % of the mothers considered the Pap test to be necessary prior to adulthood if their daughters are sexually active, 34.0 % considered it necessary in adulthood, 14.3 % considered it necessary after their daughters are married, and 12.0 % considered it to be decision for their daughters to make. Regarding the combination of HPV vaccination with the Pap test, 60.1 % of the mothers considered that their daughters need to undergo the Pap test prior to adulthood regardless of whether or not they themselves had received the HPV vaccination. Finally, 69.2 % of the mothers responded that they intended to recommend the Pap test to their daughters (Table 2).

**Table 2:** Awareness of mothers about methods for preventing cervical cancer in their daughters and about the Pap test (total *n* = 1581; missing values excluded)

	Mean ± SD or *n* (%)
1) How important are the following methods in preventing cervical cancer in your daughter? (range, 0–100)	
1. Initiating and undergoing the Pap test regularly	78.4 ± 21.4
2. Abstinence from sexual intercourse until adulthood	73.3 ± 27.9
3. Preventing sexually transmitted disease if sexually active	74.3 ± 28.5
4. Using condoms regularly	75.2 ± 26.6
5. Receiving an HPV vaccination	85.2 ± 16.6
2) When is the most suitable period for your daughter to undergo her first Pap test?	
1. Prior to adulthood if she is sexually active	595 (38.5)
2. In adulthood regardless of her sexual activity	538 (34.0)
3. After she marries	221 (14.3)
4. My daughter can decide for herself	190 (12.0)
3) If your daughter has already received HPV vaccination and is sexually active, does she also need to undergo the Pap test?	
1. The Pap test is not necessary because she has already received the HPV vaccination	18 (1.2)
2. I don’t know	312 (20.5)
3. She needs to undergo the Pap test in adulthood	277 (18.2)
4. She needs to undergo the Pap test prior to adulthood even though she has already received the HPV vaccination	914 (60.1)
4) Do you intend to recommend the Pap test to your daughter if it is considered necessary?	
Yes	1076 (69.2)
No	490 (30.9)

1) was measured prior to giving information about the Pap test and the vaccination against HPV for early screening of cervical cancer, 2) 3) 4) were measured after giving information

### Health beliefs of mothers about preventing cervical cancer in their daughters, including regarding the Pap test

Six variables of the HBM comprising 28 items were applied to examine the health beliefs of the mothers. Regarding the risks of cervical cancer in their daughters, the item of “Cervical cancer is a serious health problem for my daughter” had the lowest score (1.98 ± 0.62), indicating the highest perceived risk. Regarding the barriers to the Pap test, the item of “My daughter would not undergo the Pap test because she would be too embarrassed to have a genital exam” had the lowest score (2.68 ± 0.74), indicating that mothers were worried the most about this. For the external pressures on their daughters undergoing the Pap test, the item of “My daughter would undergo the Pap test if she heard or read something in the newspaper or in a television or radio program” had the lowest score (2.23 ± 0.64), indicating that mothers considered this to be the strongest contributing factor. Regarding the awareness of the Pap test, there was no significant difference between the scores for the items related to the age of their daughters when undergoing the Pap test (2.37 ± 0.82) and the frequency of undergoing the Pap test (2.40 ± 0.73). The 3 items related to the benefits of the Pap test had the lowest scores among the 28 items, indicating that the mothers did not agree with their beneficial effects on their daughters undergoing the Pap test. Regarding the necessity of the Pap test, the item “If my daughter does not have a child, she would not need the Pap test” had the highest score (3.02 ± 0.54), indicating that the mothers agreed the most with this statement (Table 3).

**Table 3:** Health beliefs of mothers about preventing cervical cancer in their daughters and the Pap test (total *N* = 1581; missing values excluded)

Health beliefs related to my daughter	Strongly agree (1)	Agree (2)	Disagree (3)	Strongly disagree (4)	
	*n* (%)				mean ± SD
Cervical cancer may lead to my daughter’s death	267 (17.0)	965 (61.4)	308 (19.6)	32 (2.0)	2.07 ± 0.67
Cervical cancer may lead to my daughter having a hysterectomy	258 (16.4)	1076 (68.6)	213 (13.6)	22 (1.4)	2.00 ± 0.60
Cervical cancer is a serious health problem for my daughter	294 (18.7)	1044 (66.5)	210 (13.4)	23 (1.5)	1.98 ± 0.62
Cervical cancer can lead to my daughter needing to receive chemotherapy or radiotherapy treatment	225 (14.4)	1113 (71.3)	212 (13.6)	12 (0.8)	2.01 ± 0.56
My daughter would be at risk of developing cervical cancer	106 (6.7)	989 (62.8)	434 (27.5)	47 (3.0)	2.27 ± 0.62
If my daughter has cervical cancer, she could die	160 (10.1)	956 (60.6)	439 (27.8)	23 (1.5)	2.21 ± 0.63
Cervical cancer is one of the most common cancers in women	161 (10.2)	982 (62.2)	416 (26.3)	20 (1.3)	2.19 ± 0.62
Subtotal: Risks of cervical cancer (7 items)					14.71 ± 2.97
My daughter would not have time to undergo the Pap test	37 (2.4)	346 (22.2)	950 (60.9)	227 (14.6)	2.88 ± 0.67
My daughter would not undergo the Pap test because she would not be treated in a health-care center	31 (2.0)	231 (14.9)	964 (62.3)	322 (20.8)	3.02 ± 0.66
My daughter would not undergo the Pap test because she would need to wait a long time to be seen	49 (3.2)	283 (18.4)	929 (60.4)	277 (18.0)	2.93 ± 0.70
My daughter would not undergo the Pap test because she would be afraid to find out if she has a cancer	62 (4.0)	332 (21.2)	949 (60.6)	222 (14.2)	2.85 ± 0.70
My daughter would not undergo the Pap test because the health-care center would only be open when she is not available	22 (1.4)	122 (7.8)	942 (60.4)	474 (30.4)	3.20 ± 0.63
My daughter would not undergo the Pap test because she would be too embarrassed to have a genital exam	88 (5.7)	493 (31.7)	801 (51.5)	172 (11.1)	2.68 ± 0.74
My daughter would not undergo the Pap test because it would be difficult to get an appointment	32 (2.1)	233 (15.0)	955 (61.5)	332 (21.4)	3.02 ± 0.67
Subtotal: Barriers to the Pap test (7 items)					20.57 ± 3.22
My daughter would undergo the Pap test if advised by a nurse or midwife	50 (3.2)	650 (41.8)	777 (49.9)	79 (5.1)	2.57 ± 0.64
My daughter would undergo the Pap test if advised by a doctor	131 (8.4)	845 (54.0)	523 (33.4)	65 (4.2)	2.33 ± 0.69
My daughter would undergo the Pap test if I would speak to her about it	99 (6.4)	577 (37.1)	786 (50.6)	92 (5.9)	2.56 ± 0.70
My daughter would undergo the Pap test if a friend or neighbor would speak to her about it	109 (7.0)	811 (51.9)	603 (38.6)	41 (2.6)	2.37 ± 0.65
My daughter would undergo the Pap test if members of my family would advise her to do it	76 (4.9)	718 (46.2)	685 (44.1)	74 (4.8)	2.49 ± 0.67
My daughter would undergo the Pap test if she heard or read something in the newspaper or in a television or radio program	143 (9.1)	949 (60.6)	438 (28.0)	36 (2.3)	2.23 ± 0.64
Subtotal: External pressures to undergo the Pap test (6 items)					14.55 ± 2.94
My daughter does not know at what age it would be necessary to first undergo the Pap test	129 (8.3)	820 (52.8)	506 (32.6)	97 (6.3)	2.37 ± 0.72
My daughter does not know how often she would need to undergo the Pap test	127 (8.1)	783 (49.8)	556 (35.4)	106 (6.7)	2.40 ± 0.73
Subtotal: Awareness of the Pap test (2 items)					4.77 ± 1.24
Getting the Pap test would not make my daughter feel good because it does not means that she would take care of her health	298 (19.0)	1090 (69.3)	176 (11.2)	8 (0.5)	1.93 ± 0.56
The Pap test could not save my daughter’s life	325 (20.7)	1045 (66.6)	175 (11.2)	24 (1.5)	1.94 ± 0.61
The Pap test would not influence my daughter’s health	473 (29.9)	1055 (66.7)	50 (3.2)	3 (0.2)	1.74 ± 0.52
Subtotal: Benefits of the Pap test (3 items)					5.61 ± 1.33
If my daughter has no symptoms, she would not need the Pap test	42 (2.7)	263 (16.6)	1092 (69.1)	184 (11.6)	2.90 ± 0.62
If my daughter does not have a child, she would not need the Pap test	18 (1.1)	158 (10.0)	1175 (74.5)	227 (14.4)	3.02 ± 0.54
If my daughter does not have intercourse, she would not need the Pap test	29 (1.8)	204 (13.0)	1117 (70.9)	225 (14.3)	2.98 ± 0.59
Subtotal: Necessity of the Pap test (3 items)					8.90 ± 1.53
Total 28 items					69.08 ± 7.15

### Factors associated with the intention of mothers to recommend the Pap test to their daughters

Table 4 lists the results for the associations between sociodemographic factors, health-beliefs variables, and the intention of mothers to recommend the Pap test to their daughters. In the univariate regression analysis, the mothers had a higher level of education (*p* < 0.01), were employed (*p* < 0.05), had talked about the HPV vaccination (*p* < 0.001) and the Pap test (*p* < 0.001) with their daughters, had themselves undergone the Pap test (*p* < 0.001), were younger at their first Pap test (*p* < 0.001), had a diagnostic history of cervical cancer (*p* < 0.01), and were more likely to have the intention to recommend the Pap test to their daughters. With regard to the health beliefs of the mothers, they perceived cervical cancer to be a smaller problem for their daughters (*p* < 0.01), had fewer difficulties with their daughters undergoing the Pap test (*p* < 0.01), had a greater awareness of their daughters undergoing the Pap test (*p* < 0.01), considered that the Pap test would be beneficial to their daughters and was more likely to be necessary (*p* < 0.001), and were more likely to have the intention to recommend the Pap test to their daughters (Table 4).

**Table 4:** Factors influencing the intention of mothers to recommend the Pap test to their daughters

			Univariate regression analysis		Adjusted regression analysis	
	Categories	*n* (%)	OR	95 % CI	OR	95 % CI
Sociodemographic factors						
Age	31–44 years	806 (51.8)	1	-		
	45–59 years	750 (48.2)	1.14	0.92–1.42		
Education	Middle or high school	892 (57.6)	1	-	1	-
	≥College	656 (42.4)	1.37	1.10–1.71**	1.52	1.08–2.13*
Employment status	Housewife	684 (44.1)	1		1	-
	Part or full time	866 (55.9)	1.27	1.01–1.57*	1.17	0.84–1.63
Monthly house income	1000–3500	729 (51.8)	1			
(Korean won × 1,000)	3501–5000	677 (48.2)	1.10	0.93–1.29		
Religion	No	563 (35.8)	1	-		
	Yes	1010 (64.2)	1.17	0.94–1.47		
Have talked about HPV with my daughter	No	1051 (67.0)	1	-	1	-
	Yes	518 (33.0)	2.01	1.57–2.58***	1.06	0.64–1.75
Have talked about the Pap test with my daughter	No	1191 (76.3)	1	-	1	-
	Yes	369 (23.6)	2.98	2.19–4.05***	2.11	1.23–3.64**
My daughter has received the HPV vaccination	No	1446 (92.8)	1	-		
	Yes	112 (7.2)	1.21	0.78–1.86		
I have undergone the Pap test	No	536 (35.2)	1	-	1	-
	Irregularly	712 (45.7)	2.55	1.82–3.57***	2.03	0.78–7.50
	Regularly	297 (19.1)	2.04	1.43–2.89***	1.98	1.30–3.03**
Age at first the Pap test	12–32 years	388 (39.8)	1		1	
	33–55 years	586 (60.2)	0.60	0.46–0.79***	0.60	0.43–0.82**
I have been diagnosed with cervical cancer	No	1442 (91.4)	1		1	-
	Yes	136 (8.6)	2.15	1.36–3.39**	1.90	0.99–3.66
Family history of cervical cancer	No	1058 (95.4)	1	-		
	Yes	73 (4.6)	1.19	0.71–2.02		
Health-beliefs variables						
Risks of cervical cancer^a^	High (7–14^b^)	758 (47.9)	1		1	
	Low (15–26^b^)	781 (50.7)	1.40	1.12–1.74**	1.10	0.79–1.52
Barriers to the Pap test^a^	High (9–20^b^)	807 (54.5)	1		1	
	Low (21–28^b^)	674 (45.5)	1.35	1.01–1.69**	1.47	1.03–2.11*
External pressure to undergo the Pap test^a^	High (6–14^b^)	733 (47.5)	1			
	Low (15–24^b^)	810 (52.5)	1.11	0.89–1.38		
Awareness of the Pap test	Low (2–4^b^)	729 (47.1)	1		1	
	High (5–8^b^)	818 (52.9)	1.44	1.24–1.67**	1.11	0.78–1.58
Benefits of the Pap test	Low (3–5^b^)	571 (36.6)	1		1	
	High (6–12^b^)	990 (63.4)	2.73	2.13–3.51***	2.25	1.55–3.25***
Necessity of the Pap test	Low (3–8^b^)	361 (22.9)	1		1	
	High (9–12^b^)	1213 (77.1)	1.33	1.14–1.56***	1.50	0.96–2.35

^a^Scored in reverse; ^b^Range of possible scores; *OR* odds ratio, *CI* confidence interval

*, *P* < 0.05; **, *p* < 0.01; ***, *p* < 0.001

The adjusted logistic regression analysis revealed that the significant health beliefs influencing the intention of mothers to recommend the Pap test to their daughters were the perceived barriers (OR = 1.47, 95 % CI = 1.03–2.11, *p* < 0.01) and benefits (OR = 2.25, 95 % CI = 1.55–3.25, *p* < 0.01). The corresponding sociodemographic factors were their level of education (OR = 1.52, 95 % CI = 1.08–2.13, *p* < 0.05), whether they talked about the Pap test with their daughters (OR = 2.11, 95 % CI = 1.23–3.64, *p* < 0.01), the regularity of undergoing the Pap test themselves (OR = 1.98, 95 % CI = 1.30–3.03, *p* < 0.01), and how old they were when they first underwent the Pap test (OR = 0.60, 95 % CI = 0.43–0.82, *p* < 0.01) (Table 4).

## Discussion

The Korean mothers of the girls included in this study considered that the Pap test was the second most important of five methods for preventing cervical cancer in their daughters. The mothers showed a favorable attitude toward their daughters undergoing the Pap test to prevent cervical cancer, regardless of whether they had received the HPV vaccination themselves, and most of the mothers (38.5 %) said that the most suitable period for Pap testing their daughters is prior to adulthood. These results could be interestingly interpreted as indicating that Korean mothers seemed to be more concerned about the level of sexuality than age with regard to protecting their daughters from cervical cancer. Most of the mothers responded positively to the intention to recommend the Pap test, but this might not reflect the real situation in the future. Nevertheless, this study found that the mothers showed a proactive attitude with respect to Pap testing for their daughters, which contrasts with the current national guideline for the onset of such testing to occur at an age of 20 years [13]. Future studies should explore whether the opinions of Korean mothers about the most appropriate time to commence Pap testing are associated with their concern about the sexuality of their daughters. Meanwhile, the present study has confirmed the assumption that the HBM of mothers regarding preventing cervical cancer in their daughters could significantly affect their intention to prevent cervical cancer in their daughters. In addition, the study had revealed the factors that are important to mothers, which are the level of education, the previous Pap test behaviors, and talking about the Pap test with their daughters.

The HBM in this study was effective at explaining the intentions of the mothers, supporting the fitness of the HPV framework for guiding cervical cancer and HPV prevention [8, 19, 26]. In particular, the significances of the perceived benefits and barriers in the HBM confirm the findings of previous studies [8, 19]. In the adjusted regression analysis, the factors that were critical to the intention of mothers to recommend the Pap test were the benefits and barriers that they perceived. This indicates that providing education to the mothers of adolescent girls will result in them being more accepting of the benefits and experiencing fewer barriers to their daughters undergoing the Pap test. Future studies should reexamine the other health beliefs that were not found to be significant in the intentions of the mothers in this study, so as to confirm their possible roles.

The most important factor with a negative impact regarding the opinions of the mothers was the feeling of embarrassment about their daughters undergoing the Pap test. This supports previous findings that Korean women commonly experience negative emotional reactions when they think about or undergo the Pap test or other types of gynecological examination [2, 27, 28]. As mentioned already in the Introduction, it has generally been recommended that the first Pap test should not be performed during the adolescent period due both to a low incidence of cervical cancer in that period and the possible psychological impact of the Pap test on young women [11, 12]. Thus, strategies for reducing the feeling of embarrassment could be designed to directly resolve the psychological burden that the Pap test can have on pubescent girls. The Pap test should be regarded as a women’s health right for preventing cervical cancer, regardless of interfering attitudes toward age and marriage that are still prevalent in Korean society [2]. It should be noted that the HPV vaccination rate of the Korean adolescent girls in this study was 7.2 %, which is lower than that of other countries in Asia; for example, in Taiwan adolescents this rate has been reported to be 20 % [21]. The lack of programs for administering the HPV vaccination to adolescent girls in Korea provided the rationale for this study of expanding the role of mothers in recognizing the importance of the Pap test beyond the HPV vaccination for adolescent girls in Korea. A national program of HPV vaccination needs to be applied to Korean girls, and the mothers in this study also considered HPV vaccination to be the most important method for preventing cervical cancer in their daughters. As mentioned above, the study result that the mothers generally had an intention to recommend the Pap test to their daughters suggests that many of the mothers were already aware of the factors that they were informed about during the survey. This further suggests that providing such information would be effective even when the message is short and condensed. It is also necessary to further identify differences in the intentions of mothers according to the level of universal or specific information they have received. While talking about the Pap test influences the intentions of mothers, only 23.7 % of the mothers in this study had talked about the Pap test with their daughters, which supports the finding of a previous qualitative study [15]. Strategies should therefore be developed that enhance communication about the Pap test and cervical cancer between mothers and daughters [15]. The level of education and the previous Pap test behaviors of the mothers influenced their intentions, which also supports the significant role that these factors have in the intentions of mothers to vaccinate their daughters against HPV [7, 16]. This means that the strategies applied should differ according to the level of education and the Pap test behavior when planning and implementing education for mothers. Only 19 % of the mothers in this study underwent regular Pap test screening, indicating that Korean women should be better informed about and empowered to undergo Pap testing regularly, which should in turn have a positive impact on their intention to prevent cervical cancer in their daughters.

The first limitation of this study relates to reporting bias, which is the possibility of the mothers responding in a socially expected way when the recommendations of the national guideline of the Pap test for early preventing cervical cancer in Korea had been presented to them. The presence of bias would mean that the reported intentions of the mothers would not correspond to the real situation or their future behaviors. The second limitation is that some of the subscales of the HBM (awareness of the Pap test and benefits of the Pap test for the daughters) showed unsatisfactory reliabilities, with Cronbach’s α values below 0.7, and unfortunately this study did not apply a self-efficacy variable in the HBM construct. The health beliefs construct utilized in this study should therefore be reevaluated, or self-efficacy should be included so as to increase the explanatory power for the perceptions of mothers about preventing cervical cancer in their daughters. The third limitation is that the findings cannot be generalized to the entire Korean population of mothers of pubescent girls.

This study represents the first quantitative research involving representative countrywide sampling that has provided a comprehensive understanding about the awareness and health beliefs of mothers related to preventing cervical cancer in their daughters in Korea. Future studies should develop an educational program for mothers aimed at preventing cervical cancer in their daughters, and evaluate the effect of such education on the perceptions of mothers and their intention to prevent cervical cancer in their daughters, whilst considering the level of education and the regularity of undergoing Pap testing among the mothers.

## Conclusions

The Korean mothers included in this study considered the HPV vaccination to be the most important method for preventing cervical cancer in their pubescent daughters, and expressed a favorable attitude toward the Pap test, with most of them having the intention to recommend the Pap test to their daughters; however, they simultaneously appeared to have a low perception of the benefits of Pap testing for their daughters. The factors influencing the intention of the mothers to recommend the Pap test were their perceptions of the barriers and benefits related to the Pap test in their daughters, their level of education, their experience of talking about the Pap test with their daughters, their age when they first had their own Pap test, and the regularity of them receiving the Pap test themselves. The results of this study indicate that in order to enhance the intention of mothers to recommend the Pap test to their daughters, health-care professionals should educate mothers about the benefits of their daughters undergoing Pap testing while also reducing the perceived barriers. It would also be helpful to encourage mothers to themselves regularly undergo Pap testing, and to promote communication about the Pap test and cervical cancer between mothers and daughters.

### Ethics

The research protocol was approved by the Institutional Review Board of Myoung-Ji University Hospital.

Informed consent to participate in this study was obtained from all participants.

### Availability of data and materials

It was considered to protect access to the data because of the sensitivity around the data derived from potentially identifiable human participants.

## Additional file

Additional file 1: Survey questionnaire (DOCX 26 kb)

## Abbreviations

CI: confidence interval
HBM: health belief model
HPV: human papillomavirus
OR: odds ratio
Pap: Papanicolau
