# Supplementary material for: The health beliefs of mothers about preventing cervical cancer and their intention to recommend the Pap test to their daughters: a cross-sectional survey
Source: BMC Public Health. 2016 May 3;16:370. doi: 10.1186/s12889-016-3037-6 (PMC4855431; doi:10.1186/s12889-016-3037-6)
Supplement: Additional file 1: — Survey questionnaire (DOCX 26 kb) [file 12889_2016_3037_MOESM1_ESM.docx]

Appendix: Survey Questionnaire

I. The following list presents possible methods for preventing cervical cancer, including the Pap test, in your daughter.

| 1. How important are the following methods for preventing cervical cancer in your daughter?  (Not important=0, Crucially important=100) | | |
| --- | --- | --- |
| 1-1. Initiating and undergoing the Pap test regularly (score: ) | |  |
| 1-2. Abstinence from sexual intercourse until adulthood (score: ) | |  |
| 1-3. Preventing sexually transmitted disease if sexually active (score: ) | |  |
| 1-4. Using condoms regularly (score: ) | |  |
| 1-5. Receiving an HPV vaccination (score: ) | |  |
| II. The following items are opinions or thoughts related to cervical cancer prevention in your daughter. How much do you agree with these items? Please answer each item from 1 to 4 as appropriate.  (Strongly agree=1, Agree=2, Disagree=3, Strongly disagree=4)  1. Getting the Pap test would not make my daughter feel good because it does not mean that she would take care of her health ( )  2. My daughter would not have time to undergo the Pap test ( )  3. My daughter would not undergo the Pap test because she would not be treated in a health-care center ( )  4. My daughter does not know at what age it would be necessary to first undergo the Pap test ( )  5. My daughter would not undergo the Pap test because she would need to wait a long time to be seen ( )  6. The Pap test could not save my daughter’s life ( ) |  |  |

7. My daughter would not undergo the Pap test because she would be afraid to find out if she has a cancer ( )

8. My daughter would not undergo the Pap test because the health-care center would only be open when she is not available ( )

9. My daughter would not undergo the Pap test because she would be too embarrassed to have a genital exam ( )

10. My daughter does not know how often she would need to undergo the Pap test ( )

11. My daughter would not undergo the Pap test because it would be difficult to get an appointment ( )

12. Cervical cancer may lead to my daughter’s death ( )

13. Cervical cancer may lead to my daughter having a hysterectomy ( )

14. Cervical cancer is a serious health problem for my daughter ( )

15. Cervical cancer can lead to my daughter needing to receive chemotherapy or radiotherapy treatment ( )

16. If my daughter has no symptoms, she would not need the Pap test ( )

17. If my daughter does not have a child, she would not need the Pap test ( )

18. If my daughter does not have intercourse, she would not need the Pap test ( )

19. My daughter would be at risk of developing cervical cancer ( )

20. If my daughter has cervical cancer, she could die ( )

21. Cervical cancer is one of the most common cancers in women ( )

22. The Pap test would not influence my daughter’s health ( )

23. My daughter would undergo the Pap test if advised by a nurse or midwife ( )

24. My daughter would undergo the Pap test if advised by a doctor ( )

25. My daughter would undergo the Pap test if I would speak to her about it ( )

26. My daughter would undergo the Pap test if a friend or neighbor would speak to her about it ( )

27. My daughter would undergo the Pap test if members of my family would advise her to do it ( )

28. My daughter would undergo the Pap test if she heard or read something in the newspaper or in a television or radio program ( )

Please read the following information on preventing cervical cancer presented by the Korea National Cancer Center and Korean Society of Gynecologic Oncology. Then please answer the following questions.

Tip: HPV infection can occur by sexual transmission, some types of high-risk HPV cause cervical cancer, and the Pap test is recommended annually for all sexually active women older 20 years and also for females younger than 20 years if there are suspicions of cervical cancer or a precancerous lesion. The vaccination against HPV was developed to prevent cervical cancer, and so receiving this vaccination is recommended before the onset of sexual activity.

III. Please select what you consider to be the most appropriate answers.

| 1. When is the most suitable period for your daughter to undergo her first Pap test? ( )  1) Prior to adulthood if she is sexually active |
| --- |
| 2) In adulthood regardless of her sexual activity |
| 3) After she marries |
| 4) My daughter can decide for herself |
| 2. If your daughter has already received HPV vaccination and is sexually active, does she also need to undergo the Pap test? ( )  1) The Pap test is not necessary because she has already received the HPV vaccination  2) I don’t know  3) She needs to undergo the Pap test in adulthood  4) She needs to undergo the Pap test prior to adulthood, even though she has already received the HPV vaccination |
| 3. Do you intend to recommend the Pap test to your daughter if it is considered necessary? ( )  1) No 2) Yes |
|  |

IV. Please provide the following demographic information.

1. How old are you? ( years)

2. What is the highest level of education that you have completed? ( )

1) Primary school 2) Middle school 3) High school 4) College or above

3. Which is your employment status? ( )

1) Housewife 2) Part-time job 3) Full-time job

4. What is the monthly income of your household? (×1,000 Korean won) ( )

1) 1,000–2,000 2) 2,001–3,000 3) 3,001–4,000 4) 4,001–5,000 5) Above 5,000

5. Do you have a religion? If so, please specify which it is. ( )

1) None 2) Christianity 3) Catholicism 4) Buddhism 5) Other

6. Have you ever talked about HPV with your daughter? ( ) 1) No 2) Yes

7. Have you ever talked about the Pap test with your daughter? ( ) 1) No 2) Yes

8. Has your daughter received the HPV vaccination? ( ) 1) No 2) Yes

9. Have you ever received the Pap test? If you did, was it on an irregular or regular basis? ( )

1) No 2) Irregular 3) Regular

10. If you have received the Pap test, what was your age when you first received it? ( years)

11. Have you ever been diagnosed with cervical cancer? ( ) 1) No 2) Yes

| 12. Do you have a family member diagnosed with cervical cancer? ( ) 1) No 2) Yes |
| --- |

Thank you very much
